# Role of Titanium Surface Topography and Surface Wettability on Focal Adhesion Kinase Mediated Signaling in Fibroblasts

**DOI:** 10.3390/ma4050893

**Published:** 2011-05-09

**Authors:** Christine J. Oates, Weiyan Wen, Douglas W. Hamilton

**Affiliations:** 1 Graduate Program of Biomedical Engineering, The University of Western Ontario, London, N6A 5B9 Ontario, Canada; E-Mail: coates@uwo.ca (C.J.O.); 2 Division of Oral Biology, Schulich School of Medicine and Dentistry, The University of Western Ontario, London, Ontario N6A 5C1, Canada; E-Mail: wwen@uwo.ca (W.W.)

**Keywords:** fibroblast, focal adhesion kinase, titanium, substratum roughness, wettability, adhesive signaling, tissue regeneration

## Abstract

Changes of titanium surface roughness and surface free energy may influence protein absorption that increases cell differentiation through activation of focal adhesion kinase related pathways. However, the influence of titanium surface roughness and hydrophilicity on fibroblast behavior is not well understood. The aim of this study was to investigate the influence of topography and hydrophilicity on fibroblast attachment, spreading, morphology, intracellular signaling, proliferation, and collagen I mRNA levels. Using a cellular FAK knockout (FAK^−/−^) model and wild-type (WT) controls, we also investigated the contribution of adhesion in fibroblasts cultured on smooth (PT), sand-blasted, large grit, acid-etched (SLA) and hydrophilic SLA topographies. Loss of FAK did not significantly affect fibroblast attachment to any surface, but SLA and hydrophilic SLA surface attenuated spreading of WT cells significantly more than FAK^−/−^ fibroblasts. Both FAK^−/−^ and WT cells formed numerous focal adhesions on PT surfaces, but significantly less on SLA and hydrophilic SLA surfaces. In WT cells, phosphorylation levels of FAK were lower on SLA and hydrophilic SLA in comparison with PT 24 h post seeding. Labeling of cells with antibodies to cortactin showed that FAK^−/−^ cells contained significantly more cortactin-rich focal adhesion in comparison with WT cells on PT surfaces, but not on SLA or hydrophilic SLA. ERK 1/2 phosphorylation was highest in WT cells on all surfaces which correlated with collagen I expression levels. We conclude that fibroblasts are sensitive to changes in surface roughness and hydrophilicity, with adhesive interactions mediated through FAK, an important modulator of fibroblast response.

## 1. Introduction

Surface topography, chemistry and surface energy of titanium dental implants (Ti) are a strong determinant governing integration of the device into the surrounding tissue beds [1,2,3,4]. It is recognized that inadequate connective tissue attachment to the transmucosal region of dental implants can lead to early device failure, and attachment of gingival tissues to the implant surface is important in the prevention of peri-implantitis [5]. However, many commercially available implants have smooth or “machined” topographies at the implant emergence profile region. Recent research has shown that rough topographies have been shown to increase soft tissue attachment and stability *in vivo* [6], suggesting that the addition of rough surfaces features to transmucosal regions may provide a competitive advantage for stable gingival attachment.

Pure Ti has a high initial surface free energy (SFE) and is hydrophilic due to passivation; formation of a titanium oxide (TiO_2_) layer. However, the TiO_2_ layer readily absorbs contaminants and becomes hydrophobic [7]. Techniques used to roughen the surface of Ti implants such as particle blasting, acid-etching, or Ti plasma spraying have also been shown to influence the SFE of the implant [8]. SLA (Sand-blasted, Large grit, Acid-etched) surfaces, currently employed on Institut Straumann AG implants, incorporate both micron and submicron features and is hydrophobic [9], largely due to contaminants. In recent years, a chemically modified SLA has been developed that has a higher SFE and produces a more hydrophilic surface [10,11]. The surface is produced by packaging under a nitrogen atmosphere, followed by storage of the implants in isotonic sodium chloride [8]. Despite being initially developed for enhancing osseointegration, both SLA and hydrophilic SLA are now generating interest as potential surfaces for the transmucosal region of dental implants, where attachment of gingival connective tissue is of great importance [12].

SLA alone have been shown to increase connective tissue attachment and development in a rat subcutaneous model [6], suggesting that the addition of rough surface features may increase connective tissue attachment. Interestingly, a recent *in vivo* study comparing SLA and hydrophilic SLA on subepithelial connective tissue attachment have shown that increased hydrophilicity of rough surfaces may enhance tissue attachment [12].

Despite these promising results *in vivo* [13], the combined influence of topography and surface wettability (hydrophilic SLA) on fibroblast physiology remains uninvestigated. We have previously shown that hydrophilic SLA topographies alter human gingival fibroblast (HGF) adhesion formation, as well as intracellular localization of extracellular signal-regulated kinase 1/2 (ERK1/2) in comparison with cells cultured on polished surfaces [14]. However, little *in vitro* research on cell response to hydrophilic SLA has been performed and those studies have been limited to osteoblasts [15,16,17,18,19,20,21]. Interestingly, these studies suggest that hydrophilic SLA may influence osteoblast behavior by changing protein absorption that directly induces differentiation through assembly of focal adhesion (FAs) and subsequent intracellular signaling cascade activation [16].

It has now been well established that FAs are important sites of signaling that regulate spreading, migration, cytoskeletal organization, cell cycle progression, gene expression and matrix fibrillogenesis [22,23,24]. Focal adhesion kinase (FAK) recruitment and phosphorylation at FA sites is responsible for the phosphorylation of several intracellular targets that modulate cell shape, spreading, proliferation, migration, and differentiation [25]. In fibroblasts, FAK is essential for transmission of TGF beta induction of cytoskeletal reorganization, matrix contraction, and fibrotic gene expression [26,27].

The focus of this study was (1) to investigate the influence of topography and surface wettability on fibroblast attachment, spreading, morphology, intracellular signaling, proliferation, and matrix organization, and (2) to assess the role of FAK in the regulation of these processes.

## 2. Materials and Methods

### 2.1. Ti Surfaces

**Figure 1:**
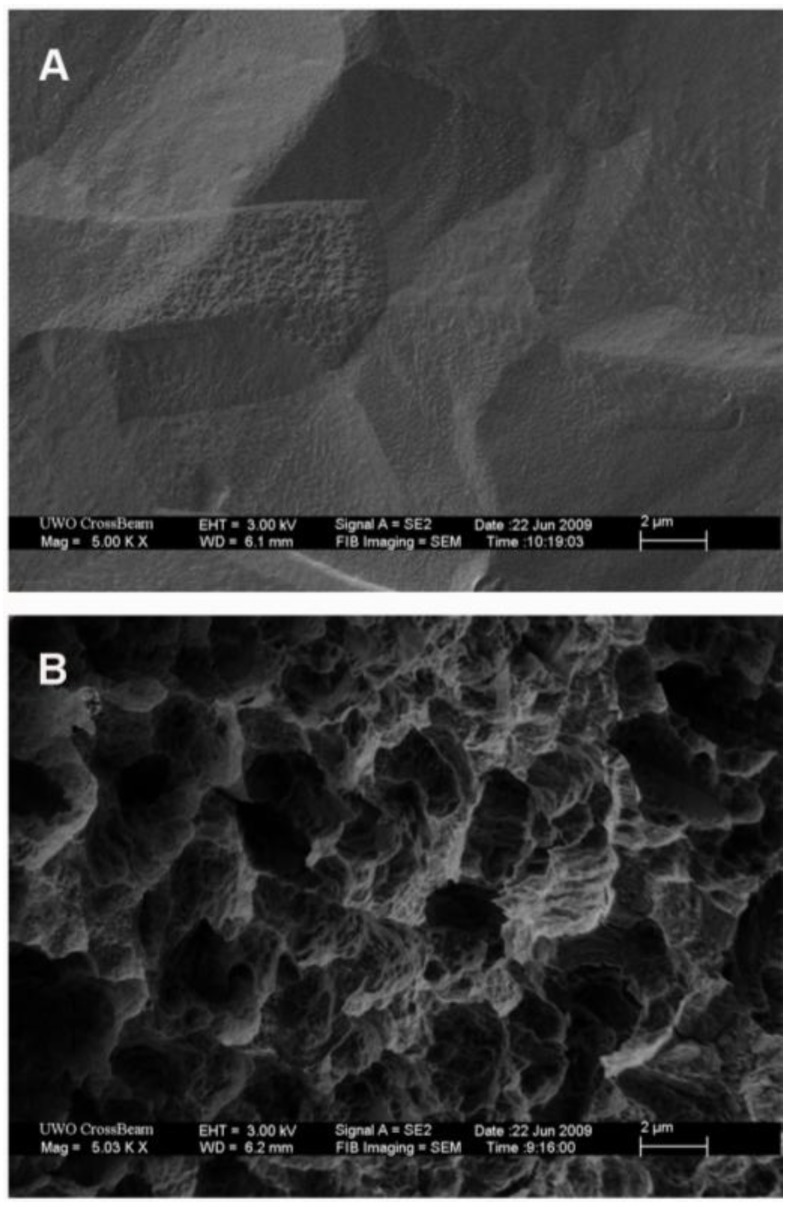
SEM images of the topographies employed in this investigation, (**A**) pickled titanium and (**B**) SLA (**S**and-blasted, **L**arge grit, **A**cid-etched).

SLA and PT Ti discs were prepared as previously described in Miron *et al.* [28]. Briefly, PT surfaces were prepared using dilute nitric acid to clean the surface, followed by washing in reverse osmosis purified water. SLA surfaces were prepared by blasting the Ti with corundum particles, followed by etching with HCl/H_2_SO_4_. Hydrophilic SLA surfaces were produced as per SLA, followed by rinsing under nitrogen protection, followed by immediate transfer to glass vials containing isotonic NaCl solution. The preparation of each surface, as well as the topographical and chemical properties of these surfaces, has been described in detail by both Rupp *et al.* 2005 [7] and Zhao *et al.* 2007 [16]. SEM images of the topographies are shown in Figure 1.

### 2.2. Cell Culture

FAK knockout (FAK KO) and wild-type (WT) embryonic fibroblasts were obtained from American Type Culture Collection (Manassas, VA, USA) and cultured as previously described [26,27]. Briefly, fibroblasts were taken from *fak (focal adhesion kinase)^+/+^* and *fak^−/−^* mice and grown in DMEM media containing 10% fetal calf serum, 2 mM L-glutamine, and antibiotics (100 units/mL penicillin and 100 µg/mL streptomycin). Cells were removed from the tissue culture plastic using a trypsin solution [0.25% trypsin (Gibco), 0.1% glucose, citrate-saline buffer (pH 7.8)].

### 2.3. Adhesion, and Spreading and Proliferation Assays

Cells were seeded on structures in 24-well plates at a density of 15,000 cells per well. Cell number was measured by staining cells with DAPI at 30 min, 2 h, and 6 h for cell adhesion and 1, 2 and 3 days for cell proliferation assays. Spreading assays were performed using our previously described assays [28,29]. Cells were fixed in 4% paraformaldehyde (PFA) and stained with DAPI. Ten fields of view were captured per sample on an AxioScope fluorescence microscope (Zeiss) and number of nuclei were counted using AxioImager software (Zeiss). Cells were thresholded using AxioImager software and the average planar area of the cells was measured. Data was expressed as average planar area ± standard error of the mean.

### 2.4. Immunofluorescence

FAK^−/−^ and WT cells were plated at a density of 25,000 cells per structure and were fixed in 4% PFA at 30 min, 2 h, 6 h, and 24 h post-seeding. Samples were then stained with rhodamine-conjugated phalloidin (Sigma-Aldrich), 4,6-diamidino-2-phenylindole dihydrochloride (DAPI) (Sigma-Aldrich), vinculin (Sigma-Aldrich), and cortactin (Millipore) antibodies, as previously described [28,30]. Images were captured from each surface on an AxioScope microscope (Zeiss) using an Axiocam digital camera and AxioImager software.

### 2.5. Western Blotting

40 μg of total protein was mixed in a 1:1 ratio with Laemelli buffer and 2-mercaptoethanol and boiled for 5 min to denature the protein. Samples were then loaded onto 10% sodium dodecyl sulfate page gels and the samples run at 40 mA for 1 h. Immunoblotting for the detection of FAK and ERK 1/2 was performed as described previously [14,30,31].

### 2.6. Taq Man Polymerase Chain Reaction

PCR was performed as previously described [28]. Total RNA was isolated using TRIZOL reagent and RNAeasy Mini kit (QIAGEN) at 7 days. Following Trizol extraction, real-time RT-PCR was performed using 15 µL final reaction volume of TaqMan’s One step Master Mix kit (Applied Biosystems). 40 ng of total RNA was used per sample well. Each sample contained pooled mRNA from trizol extractions collected from 3 Ti surfaces. Specific primer and probe sequences for collagen type I were employed. All samples were assayed in triplicate and 3 independent experiments were performed. The 2^−ΔΔCt^ method was used to calculate gene expression levels relative to 18 S and normalized to control cells (PT). Data were log-transformed prior to analysis by one-way ANOVA with Bonferroni *post-hoc* testing, using Graphpad Software v.4 (Graphpad Software, La Jolla, CA, USA).

### 2.7. Statistical Analysis

For adhesion and proliferation experiments, three independent experiments were performed each with three replicates for each condition per experiment. Data were analyzed for statistical significance using 2-way analysis of variance (ANOVA) with Bonferroni test. In spreading experiments, a minimum of fifteen cells from each treatment group was counted, with three replicates per experiment, and three independent experiments performed. Data were analyzed for statistical significance in GraphPad Software v.4 (Graphpad Software, La Jolla, CA) using 2-way analysis of variance (ANOVA) with Bonferroni post-test.

## 3. Results

### 3.1. Fibroblast Attachment 

**Figure 2:**
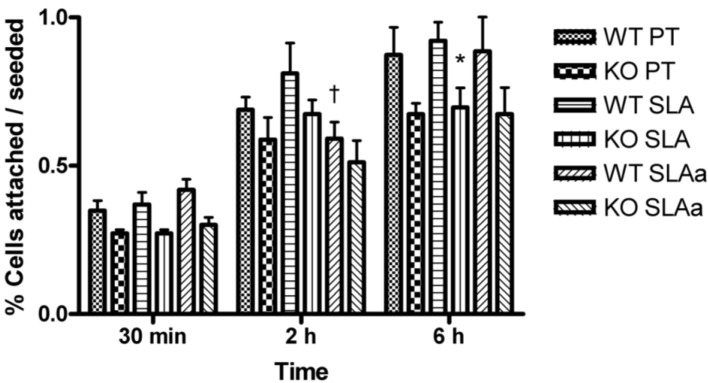
Influence of substratum topography and hydrophilicity on attachment of FAK^−/−^ and WT fibroblasts to PT, SLA and hydrophilic SLA at 30 mins, 2 and 6 h post-seeding. Data were analyzed for statistical significance using 2-way analysis of variance (ANOVA) with Bonferroni test. In (**A**), † = significance (*p* < 0.05) between cells on SLA and hydrophilic SLA and * = significance (*p* < 0.05) between KO *vs*. WT.

FAK^−/−^ and WT fibroblasts attached to PT, SLA and hydrophilic SLA surfaces at a similar rate and number (Figure 2). After 6 h, there were no statistical differences in the percentage of cells attached to each surface type, although WT cell attachment was significantly higher than FAK^−/−^ cells on SLA (*p* < 0.05).

### 3.2. Focal Adhesion Formation, Spreading and FAK Phosphorylation

**Figure 3:**
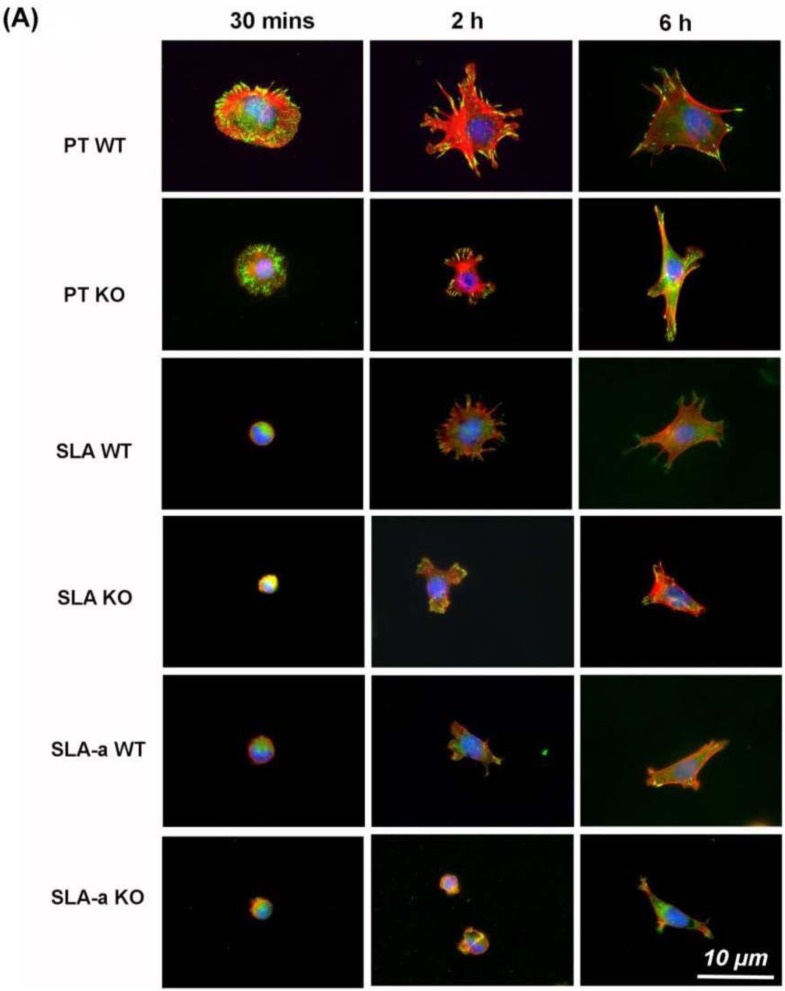

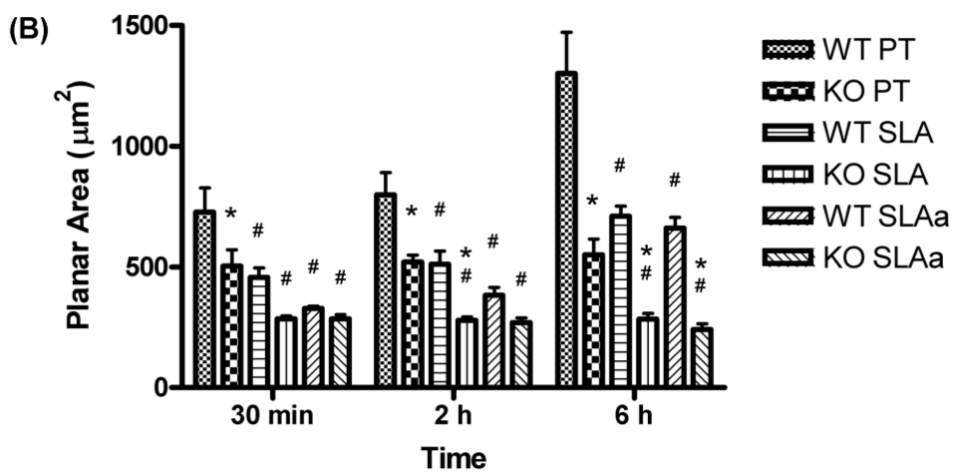
Influence of substratum topography and hydrophilicity on spreading and adhesion formation of FAK^−/−^ and WT fibroblasts. In (**A**), cells were labeled with antibodies to vinculin (green) to visualize focal adhesions and counterstained with rhodamine-phalloidin (red) to visualize F-actin; (**B**) Spreading (planar area) of fibroblasts cultured on PT, SLA and hydrophilic SLA 30 mins, 2 and 6 h post-seeding. Data were analyzed for statistical significance using 2-way analysis of variance (ANOVA) with Bonferroni test. * = significance (*p* < 0.05) between KO *vs.* WT, and # = significance between SLA and hydrophilic SLA *vs*. PT (*p* < 0.05).

**Figure 4:**
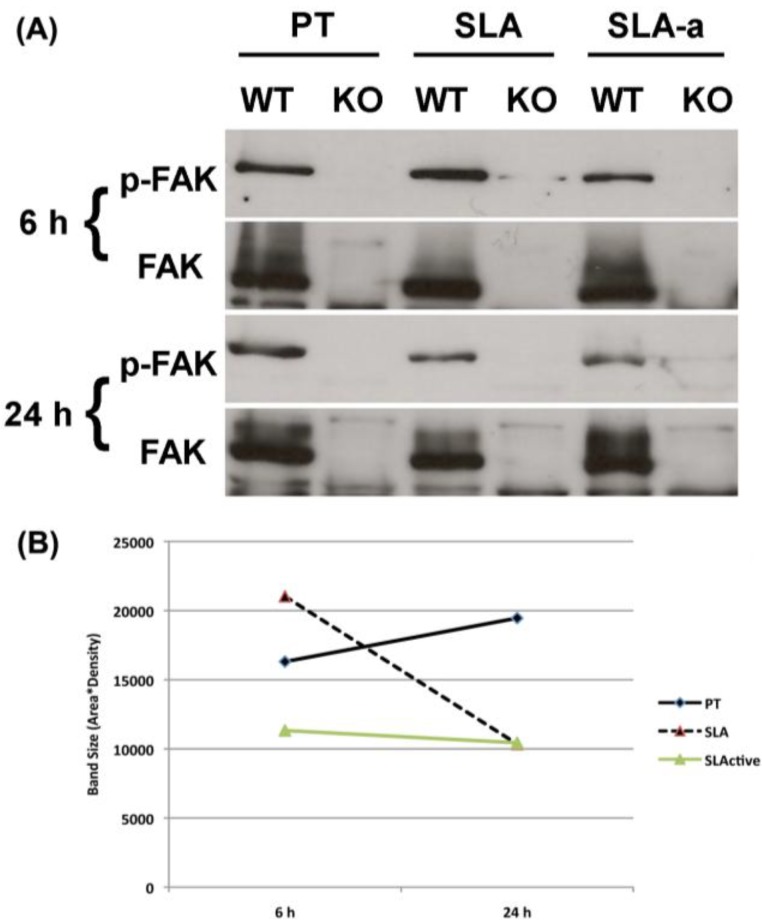
Influence of substratum topography and hydrophilicity on phosphorylated and total FAK in FAK^−/−^ and WT fibroblasts cultured on PT, SLA and hydrophilic SLA at 6 and 24 h post seeding. (**A**) Representative Western blot image. Note the reduction in phosphorylated FAK in WT cells cultured on SLA and Hydrophilic SLA. (**B**), analysis of FAK band size/density using imageJ software.

We next investigated FA formation using immunolabeling for the FA-associated protein vinculin. Distinct focal adhesions were visible at the periphery of both WT and KO cells on PT 30 min post-seeding (Figure 3A), with a reduction in number at 2 and 6 h. On SLA, both FAK^−/−^ and WT cells showed fewer FAs at 30 mins. At 2 h, WT cells cultured on SLA extended numerous microspikes, which resulted in formation of vinculin associated cell extensions at 6 h. FAK^−/−^ cells failed to develop microspikes on SLA and the number and size of FAs was reduced at both 2 and 6 h. Both FAK^−/−^ and WT cells cultured on Hydrophilic SLA contained very few vinculin-containing FAs. Both FAK^−/−^ and WT cells had a significantly lower planar area on both SLA and hydrophilic SLA surfaces, compared to PT, at 30 mins, 2 and 6 h (*p* < 0.05) (Figure 3B). At 6 h, on all surfaces, FAK^−/−^ cells exhibited a significantly lower planar area than WT cells (*p* < 0.05). As both FAK^−/−^ and WT cells showed a reduction in the number of FAs on SLA and hydrophilic SLA surfaces, we investigated whether FAK phosphorylation was reduced in WT cells using Western blotting (Figure 4A,B). At 6 h, WT cells cultured on SLA exhibited similar levels of phospho-FAK on all surfaces (Figure 4A). However, at 24 h, cells cultured on PT surfaces had higher levels of phospho-FAK than WT cells cultured on either SLA or hydrophilic SLA.

### 3.3. F-actin Arrangement and Cortactin Localization

**Figure 5:**
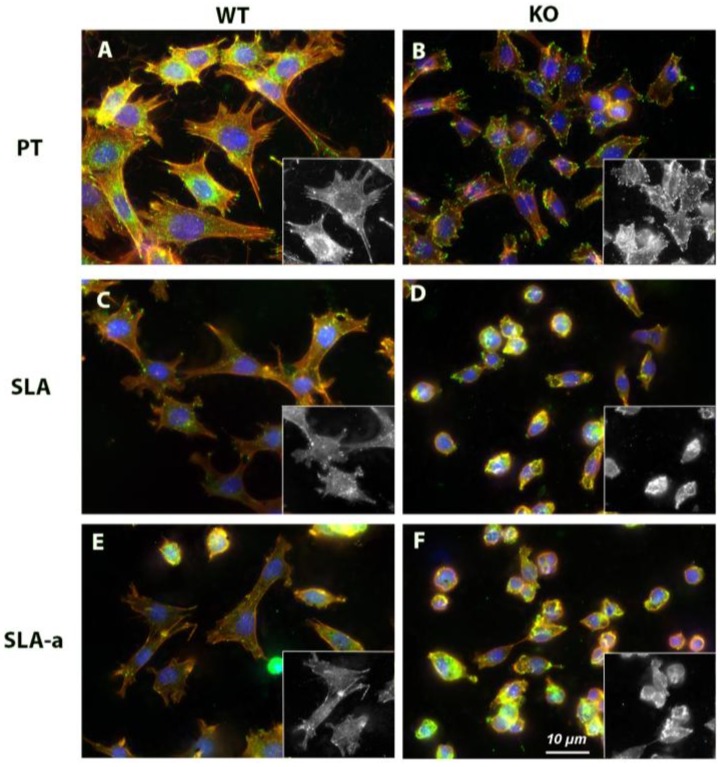
Influence of substratum topography and hydrophilicity on cortactin localization in FAK^−/−^ and WT fibroblasts. FAK^−/−^ and WT fibroblasts were cultured on PT, SLA (not shown) and hydrophilic SLA for 24 h and labeled with antibodies to cortactin (green), rhodamine-phalloidin for F-actin (red) and DAPI to visualize the nucleus (blue).

As F-actin stressfibers insert into FAs, we next assessed whether the changes in the spreading and FA formation in WT and FAK^−/−^ on PT, SLA and Hydrophilic SLA correlated with changes in cytoskeletal organization (Figure 5). On PT surfaces, WT cells formed numerous F-actin stressfibers, which were stabilized by the F-actin binding protein cortactin (Figure 4). FAK^−/−^ fibroblasts also formed stressfiber bundles on PT, but exhibited significantly more cortactin plaques at the cell periphery. On SLA and hydrophilic SLA, both WT and FAK^−/−^ fibroblasts did not form stressfiber bundles and cortactin labeling was reduced, with cortactin appearing to diffuse throughout the cytoplasm.

### 3.4. Activation of ERK 1/2

**Figure 6:**
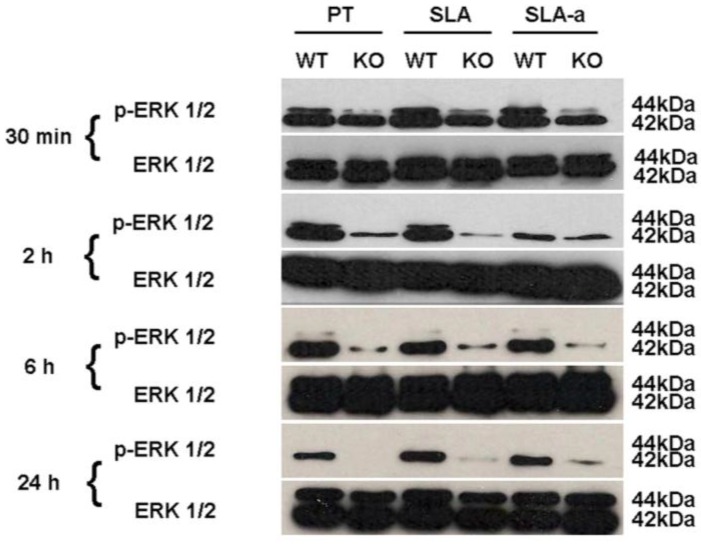
Influence of substratum topography and hydrophilicity on ERK 1/2 phosphorylation in FAK^−/−^ and WT fibroblasts. Western blotting using specific antibodies to phosphorylated and total ERK 1/2 at 30 mins, 2, 6 and 24 h post seeding. ERK 1 is 44 kDa (upper band) and ERK 2 is 42 kDa (lower band).

As phosphorylation of ERK 1/2 is a common downstream target of FAK activation [23], we determined by Western blotting whether surface topography altered levels of ERK 1/2 phosphorylation in FAK^−/−^ and WT cells (Figure 6). At 30 min, post seeding, WT cells cultured on SLA surfaces exhibited higher levels of phospho-ERK 1/2 on all surfaces in comparison with FAK^−/−^ cells. At 2 hrs, phospho-ERK 1/2 was highest in WT cells cultured on PT and SLA. In FAK^−/−^ cells, only ERK 2 phosphorylation levels were present on all surfaces, with similar levels of phospho-ERK 1/2 still evident in WT cells on hydrophilic SLA. At 6 h, phospho-ERK 1/2 declined in WT cells on all surfaces and at 24 h only ERK 2 remained phosphorylated on all surfaces. In FAK^−/−^ cells at 6 h, only phospho-ERK 2 was detected on all surfaces, dropping to undetectable levels on PT and SLA, but not hydrophilic SLA at 24 h.

### 3.5. Proliferation

**Figure 7:**
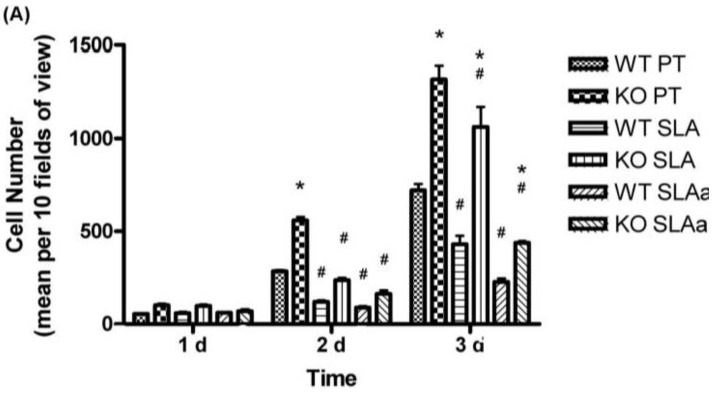

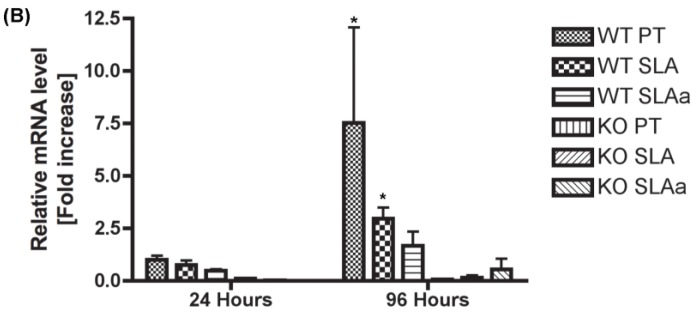
Influence of substratum topography and hydrophilicity on cell proliferation and type I collagen mRNA levels in FAK^−/−^ and WT fibroblasts. (**A**) To assess proliferation, cells were cultured on PT, SLA and hydrophilic SLA for 1, 2 and 3 days and number of cells counted as described in Section 2.3. In (**B**), fibroblast differentiation, we measured the mRNA levels of type I collagen mRNA using realtime Taqman PCR. Data were analyzed for statistical significance using 2-way analysis of variance (ANOVA) with Bonferroni test. In (**A**) * = significance (*p* < 0.05) between KO *vs.* WT, and # = significance between SLA and hydrophilic SLA *vs.* PT (*p* < 0.05). In (**B**) * = significance (*p* < 0.05) between WT at 24 and 96 h on PT and SLA.

To further assess the role of FAK in fibroblast physiology, we quantified proliferation of WT and FAK^−/−^ fibroblasts at 1, 2 and 3 d post seeding. FAK^−/−^ fibroblasts number was significantly higher than WT on all surfaces after 2 and 3 days post seeding (Figure 7A). WT and FAK^−/−^ fibroblasts number was always significantly higher on PT surfaces than on SLA or hydrophilic SLA. After 3 days, significantly more WT and FAK^−/−^ fibroblasts were present on SLA than hydrophilic SLA.

### 3.6. Fibroblast Differentiation—Collagen I Production

As FAK and substratum topography have been shown to influence cell differentiation, we assessed whether FAK deletion influenced mRNA levels of collagen type I in fibroblasts cultured on PT, SLA and hydrophilic SLA (Figure 7B). At 24 h, there was no significant difference in collagen type I mRNA levels in WT and FAK^−/−^ fibroblasts on any tested surface. At 96 h, collagen type I mRNA levels were significantly higher in WT fibroblasts cultured on PT and SLA surfaces (*p* < 0.05) compared with hydrophilic SLA. There was no significant difference in collagen type I mRNA levels in FAK^−/−^ fibroblasts on any surface.

## 4. Discussion

In this study, we assessed two different topographies (PT *vs.* SLA) as well as the influence of surface hydrophilicity on fibroblast behavior and whether FAK was required for adhesion, spreading, intracellular signaling, proliferation, differentiation, and matrix organization by fibroblasts cultured on these surfaces. We utilized FAK^−/−^ mouse embryonic fibroblasts isolated from knockout mice and fibroblasts from embryonic wild-type mice as controls. The cell lines derived from FAK^–/–^ mice have been shown defects in spreading and migration compared with the wild-type mice [32] and the importance of FAK in cell function is evident from the embryonic lethality associated with deletion [33]. However, we are aware of certain limitations on the use of these cells. PYK-2, a FAK homolog is significantly upregulated likely to compensate for a lack of FAK, but it is not sufficient to rescue the phenotype of the cells [34]. In addition, it is known that the cell cycle checkpoint gene p53 is inactive [35]. However, we have carefully taken these issues into account in the interpretation of our data.

Level of roughness and hydrophilicity significantly reduced the spreading of WT fibroblasts, which was compounded further by the deletion of FAK. Indeed on hydrophilic SLA, WT cells were similar in appearance to FAK^−/−^ cells, which correlated with reduced FA formation and with a reduction in levels of phospho-FAK in WT cells. These findings are in direct contrast to Lai *et al.* 2009, who demonstrated that human alveolar osteoblasts spread more on hydrophilic SLA than SLA, with an increase in FAK mRNA levels at 6 h on Hydrophilic SLA [19]. The difference in findings could be accounted for by the fact we assessed phosphorylation of FAK as opposed to mRNA levels, as well as the difference in cell type. As both FAK^−/−^ and WT fibroblasts spread less and form fewer adhesions on SLA and hydrophilic SLA surface in comparison with PT, we assessed the stability of the FAs through immunolabeling with the F-actin binding protein cortactin. Cortactin is a substrate for both tyrosine and serine/threonine kinases, with the former being involved in FA stability [36]. Using an antibody specific for tyrosine phosphorylation of cortactin, we observed on PT surfaces, FAK^−/−^ fibroblasts form significantly more cortactin rich plaques in the cell periphery in comparison with WT cells. It is possible that FAK^−/−^ cells, which form less mature adhesions, may attempt to stabilize adhesions formed through increased recruitment of cortactin to the cell periphery and subsequently into FAs. This is in agreement with previous studies that have shown that siRNA knockdown of FAK in fibroblasts results in inappropriate lamellapodia formation, characterized by enrichment of cortactin within the extensions [35]. As both FAK^−/−^ and WT cells show a reduced number of FAs on SLA and hydrophilic SLA, it is clear that both roughness and hydrophilicity influence assembly of FAs. Inability of cells to stabilize adhesions would likely significantly inhibit activation of downstream signaling cascades regulating proliferation and differentiation.

We have previously demonstrated that altered patterns of adhesion formation directly results in changes in ERK 1/2 signaling in gingival fibroblasts cultured on SLA topographies [14]. ERK 1/2 signaling has been implicated in regulation of proliferation [37] and is known to be an important signaling cascade in fibrosis. ERK 1/2 phosphorylation remained higher in WT cells on all surfaces, demonstrating that FAK in part regulates ERK 1/2 phosphorylation as has been previously observed [38]. Interestingly, proliferation rates of FAK^−/−^ fibroblasts were significantly lower on SLA and hydrophilic SLA surfaces, despite the loss of the cell cycle checkpoint protein, p53. Reductions in osteoblast proliferation on SLA and hydrophilic SLA have been previously reported [16], showing that surface roughness is likely to exert the largest effect on cell division. At 24 hrs phosphorylated ERK 2 levels remain high in WT cells on all surfaces, which correlated with an increase in collagen mRNA levels. Ablation of ERK 2 with siRNA has been demonstrated to inhibit collagen type 1 synthesis [39]. In the absence of FAK, induction of collagen type I mRNA synthesis was negligible, showing that in the absence of FAK, substratum topography and hydrophilicity have no influence on induction of collagen type I gene expression in fibroblasts. Our findings that hydrophilic SLA surfaces reduce fibroblast differentiation is in contrast to many of the studies on osteoblasts, which respond to Hydrophilic SLA by reducing proliferation and increasing expression of biomineralization markers in comparison with cells cultured on SLA [15,16,20]. Qu *et al.* concluded that the influence of free surface energy (hydrophilic SLA) on cell response was likely to be an indirect effect, with initial hydrophilicity influencing osteogenic protein and amino acid absorption on the topographies more so than on initially hydrophobic surfaces (PT and SLA) [15].

Overall our results suggest that *in vitro*, hydrophilic SLA impairs fibroblast function by reducing adhesion formation, spreading, ERK phosphorylation and collagen type I expression in comparison with cells cultured on either PT or SLA surfaces. Moreover, our results with FAK null fibroblast show that activation of FAK is essential for many of these cellular functions. Our results are in direct contrast with *in vivo* pilot studies using dogs, which demonstrated hydrophilic SLA topographies enhance connective tissue attachment at the transmucosal region of an implant in comparison with SLA [12]. Well-attached subepithelial connective tissue exhibiting collagen fibers that started to extend and attach partially perpendicular to the implant surface were evident surrounding hydrophilic SLA. In comparison, SLA implants had a dense zone of collagen fibers running parallel to the implant surface. Clearly protein absorption in the *in vivo* environment will be significantly different from the *in vitro* cell culture system, which could significantly influence how fibroblasts respond to the titanium surfaces. It is possible *in vivo* protein absorption favors profibrogenic proteins and *in vitro* different proteins attach from serum that do not induce beneficial fibroblast behaviors. Further evidence for this comes from that fact that PT surfaces, which have lower surface energy in comparison with either SLA or hydrophilic SLA, elicit the largest fibrogenic response.

## 5. Conclusions

It is only now that we are beginning to recognize and appreciate the functional significance of changes in adhesion complex formation in cells, particularly when cells are grown on surfaces with changes in substratum topography and hydrophilicity. Moreover, it adds further evidence that SFE, hydrophilicity and subsequent protein absorption, plays a very significant role in soft tissue attachment to titanium. By understanding how biomaterial properties influence protein absorption and cell behavior at the molecular level, more suitable biomaterials can be developed to enhance tissue regeneration *in situ* or in lab-based tissue engineering applications.
